# OView-AI Supporter for Classifying Pneumonia, Pneumothorax, Tuberculosis, Lung Cancer Chest X-ray Images Using Multi-Stage Superpixels Classification

**DOI:** 10.3390/diagnostics13091519

**Published:** 2023-04-23

**Authors:** Joonho Oh, Chanho Park, Hongchang Lee, Beanbonyka Rim, Younggyu Kim, Min Hong, Jiwon Lyu, Suha Han, Seongjun Choi

**Affiliations:** 1 Department of Mechanical System Engineering, Chosun University, Gwangju 61452, Republic of Korea; oh@theotom.com; 2 OTOM, Co., Ltd., Gwangju 61042, Republic of Korea; rbbnk@theotom.com (B.R.); onetop@theotom.com (Y.K.); 3 Department of Radiology, Soonchunhyang University Cheonan Hospital, Cheonan 31151, Republic of Korea; parksam-f@hanmail.net; 4 Haewootech Co., Ltd., Busan 46742, Republic of Korea; hclee@haewootech.co.kr; 5 Department of Computer Software Engineering, Soonchunhyang University, Asan 31538, Republic of Korea; mhong@sch.ac.kr; 6 Division of Respiratory Medicine, Department of Internal Medicine, Soonchunhyang University Cheonan Hospital, Cheonan 31151, Republic of Korea; 78214@schmc.ac.kr; 7 Department of Nursing, Soonchunhyang University Cheonan Hospital, Cheonan 31151, Republic of Korea; jasmin720@schmac.ac.kr; 8 Department of Otolaryngology-Head and Neck Surgery, Cheonan Hospital, Soonchunhyang University College of Medicine, Cheonan 31151, Republic of Korea

**Keywords:** deep learning, EfficientNet, pneumonia, pneumothorax, tuberculosis, lung cancer

## Abstract

The deep learning approach has recently attracted much attention for its outstanding performance to assist in clinical diagnostic tasks, notably in computer-aided solutions. Computer-aided solutions are being developed using chest radiography to identify lung diseases. A chest X-ray image is one of the most often utilized diagnostic imaging modalities in computer-aided solutions since it produces non-invasive standard-of-care data. However, the accurate identification of a specific illness in chest X-ray images still poses a challenge due to their high inter-class similarities and low intra-class variant abnormalities, especially given the complex nature of radiographs and the complex anatomy of the chest. In this paper, we proposed a deep-learning-based solution to classify four lung diseases (pneumonia, pneumothorax, tuberculosis, and lung cancer) and healthy lungs using chest X-ray images. In order to achieve a high performance, the EfficientNet B7 model with the pre-trained weights of ImageNet trained by Noisy Student was used as a backbone model, followed by our proposed fine-tuned layers and hyperparameters. Our study achieved an average test accuracy of 97.42%, sensitivity of 95.93%, and specificity of 99.05%. Additionally, our findings were utilized as diagnostic supporting software in OView-AI system (computer-aided application). We conducted 910 clinical trials and achieved an AUC confidence interval (95% CI) of the diagnostic results in the OView-AI system of 97.01%, sensitivity of 95.68%, and specificity of 99.34%.

## 1. Introduction

There are several different types of diseases that prevent lungs from functioning properly [1]. The diseases that affect the alveoli region of the thoracic region are pneumonia, tuberculosis, and lung cancer [2]. Pneumonia is an inflammation that occurs in the subcellular region of the lungs, especially the alveoli (air sac) [3]. Tuberculosis bacteria are classified as infectious, acute, or chronic diseases with the lungs being infected. Pulmonary tuberculosis can be diagnosed as the presence of cheese-like granuloma mass in the lungs from chest X-ray images, and this symptom is called cavitation [4]. Lung cancer affects uncontrolled cell growth in the tissues of the lung [2]. In South Korea, lung cancer is the third highest among all cancers, as reported by the annual report of the Korean National Cancer Registration Statistics [5]. Air pneumothorax, or pneumothorax, refers to a disease in which air leaks through a hole in the lungs and air fills the pleural cavity. As the amount of air in birds increases, the lungs do not function normally [2]. Pneumothorax affects partial or complete lung collapse [6]. Those abovementioned lung diseases can be primarily and commonly assessed through chest radiography by professional radiologists. An early screening of the disease and regular monitoring of the changes in the disease can reduce the life-threatening consequences.

Chest X-ray images are non-invasive chest radiographs which are easy and economical for lung abnormality inspection. They assist radiologists in screening and diagnosing the overall findings of the chest in brief. However, the manual examination of a chest X-ray image is highly prone to error and is time-consuming for the person conducting the task. Therefore, computer-aided application helps to fasten and increase the accuracy of this examination.

In recent years, with the rapid innovation of artificial intelligence in computer vision, deep learning has been deployed in medical image analysis, including image classification, segmentation, detection, and localization tasks [2,7,8]. The computer-aided solution is one of the medical image analyses which use chest X-ray images to assist with the diagnosis of lung disease. Unlike traditional machine learning methods, deep learning can extract potential features from chest X-ray images and then classify them in end-to-end learning without user intervention. However, chest X-ray images pose a challenge for deep learning techniques due to their high inter-class similarities and low intra-class variant abnormalities, especially in terms of the complex nature of radiographs and the complex anatomy of the chest. For instance, pneumonia, tuberculosis, and lung cancer occur within the alveoli region of the thoracic region, for which it is hard to identify the different patterns of their disorders. Additionally, most deep learning research of the image classification task focuses on natural image modalities (RGB images). Since a chest X-ray image is a grayscale image, it results in more challenges for the deep learning method. Moreover, all chest X-ray images were scanned only at the chest area in which the output images have the same pattern of the chest anatomy.

Convolutional neural network (CNN) has proved its promising performance in 2D image classification tasks. Early state-of-the-art CNN models such as AlexNet, VGG, ResNet, DenseNet, MobileNet, InceptionNet, XceptionNet, and EfficientNet, to name a few, have proved their significant performances trained on the ImageNet dataset. The ImageNet dataset consists of thousands of classes and millions of images. “Training from scratch” on a large dataset requires a much higher computation cost and much more time. Therefore, a “transfer learning” technique has played an important role in closing the gap between the computation cost and the significant performance.

Therefore, in this study, we propose a fine-tuned deep-learning-based solution to classify multi-chest infections using chest X-ray images to improve the efficiency and effectiveness of diagnosis using the computer-aided application (namely, OView-AI system).

Our main contributions are as follows:

- We exploited the EfficientNet B7 [9] with the pre-trained weights of ImageNet trained by Noisy Student [10] as a backbone model; this was then followed by proposed fine-tuning layers and hyperparameters;
- Our method is end-to-end learning which extracts the potential features of a chest X-ray image directly and then applies the Softmax function to generate the different predicted class probabilities;
- Our experiment was conducted on a dataset which collected data from Soonchunhyang University Hospital from May 2022 to July 2022 and were labeled by professional radiologists of Soonchunhyang University Hospital after de-identification;
- We conducted an inference, of which the inferencing results were evaluated and confirmed by professional radiologists of Soonchunhyang University Hospital;
- Our study was conducted on the trend of major lung diseases occurring in South Korea, such as pneumonia, pneumothorax, tuberculosis, and lung cancer.

## 2. Related Works

Table 1 lists recently published related papers conducted on the classification of lung diseases. The list is sorted by the largest number of classes. Asif et al. [11] conducted the transfer learning technique with the pre-trained weights of VGG-16 to classify two lung diseases (COVID-19, viral pneumonia, and normal). The method achieved an accuracy of 97.84%. Hamwi et al. [12] conducted the transfer learning technique with the pre-trained weights of a merged model of VGG-16 and DenseNet-201 to classify one lung disease (COVID-19 and normal). The method achieved an accuracy of 99.73%. Hong et al. [13] used the transfer learning technique with the pre-trained weights of EfficientNet B7 to classify three lung diseases (Pneumonia, pneumothorax, tuberculosis, and normal). The method achieved an accuracy of 96.10%. Hu et al. [14] conducted training from scratch using VGG-16 architecture to classify two lung diseases (COVID-19, non-COVID-19 pneumonia, and normal). The method achieved an AUC-ROC of 97.80%. Kim et al. [15] utilised the transfer learning technique with the pre-trained weights of EfficientNet v2M to classify three lung diseases (pneumonia, pneumothorax, tuberculosis, and normal). The method achieved an accuracy of 82.20%. Malik et al. [16] conducted training from scratch using the residual network and dilated convolution to classify four diseases (pneumonia, pneumothorax, tuberculosis, COVID-19, and normal). The method achieved an accuracy of 99.39%. Manalakis et al. [17] used the transfer learning technique with the pre-trained weights of a merged model of DenseNet-121 and ResNet-50 to classify three lung diseases (COVID-19, tuberculosis, pneumonia, and normal). The method achieved an AUC-ROC of 95.00%. Shamrat et al. [18] conducted transfer learning with the pre-trained weights of VGG-16 to classify nine lung diseases (COVID-19, effusion, tuberculosis, pneumonia, lung opacity, mass, nodule, pneumothorax, pulmonary fibrosis, and normal). The method achieved an accuracy of 98.89%. Showkatian et al. [19] conducted training from scratch using the CNN model to classify one lung disease (tuberculosis and Normal). The method achieved an accuracy of 87.00%. Xu et al. [20] conducted training from scratch using DenseNet architecture to classify two lung diseases (tuberculosis, diseases but non-TB, and normal). The method achieved an accuracy of 99.10%.

## 3. Methods

### 3.1. Dataset

Chest X-ray images, such as normal, pneumonia, pneumothorax, tuberculosis and lung cancer, were collected from Soonchunhyang University Hospital X-ray Database from May 2022 to July 2022. In total, 5 groups of 1000 dicom image files were collected after confirmation by pulmonary and radiologic specialists at Soonchunhyang University Hospital. Four disease groups were collected at a 1:1 ratio according to disease severity, such as mild and severe groups. The disease severity was anonymized, and the readers (pulmonary and radiologic specialists) were blinded to the clinical data aside from the known diagnosis of pneumonia, pneumothorax, tuberculosis, and lung cancer in all patients. The criteria for disease severity are as follows: Each lung in pneumonia was divided into an upper and a lower zone by equally dividing the hila into upper and lower segments in the craniocaudal dimension. The extent of disease in each of the four zones was scored on a 0–3 scale (0 = no disease, 1 = less than one-third of the zone, 2 = one-third to two-thirds of the zone, and 3 = more than two-thirds of the zone). The severity of disease in each zone was also scored on a 0–3 scale (0 = normal lung parenchyma, 1 = airspace opacification that does not obscure the bronchovascular markings, 2 = airspace opacification that partially obscures the bronchovascular markings, and 3 = complete opacification of the alveolar and interstitial spaces). The extent and severity scores were given as equal weights and were summed over the four lung regions (right upper, right lower, left upper, and left lower) to create a composite score for each CXR ranging from 0 (normal) to 24 [21].

The severity of tuberculosis was calculated by Timika score [22]. The CXR was divided into six zones of a roughly similar size with two horizontal lines and for each zone; the percentage area that showed active disease (consolidation, nodules) involvement was estimated depending on the visual estimation of the extent of opacification (5 or 10–100% in 10% increments), and the CRX score = proportion of total lung affected (%) +40 if cavitations present.

The severity of pneumothorax was calculated by the American College of Chest Physicians guidelines (2001). We measured the size of pneumothorax from thoracic cupola to the lung apex.

**Figure 1:**
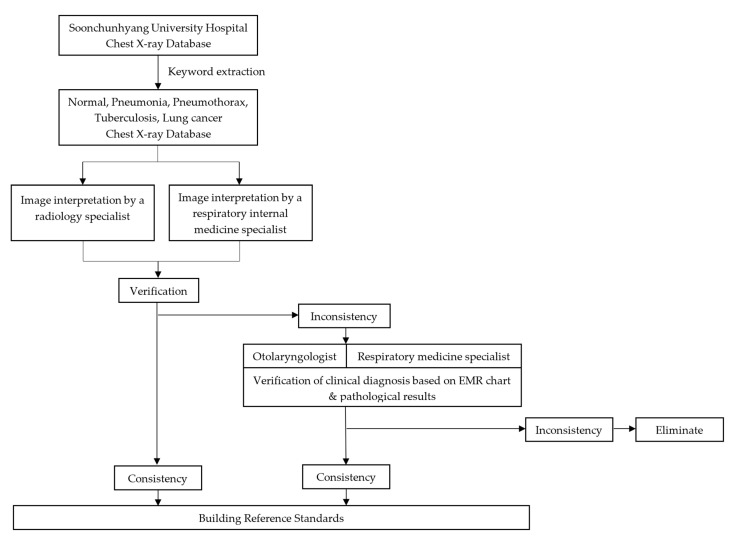
Overall study flow of dataset collecting and labeling for the multi-classification for lung diseases.

**Figure 2:**
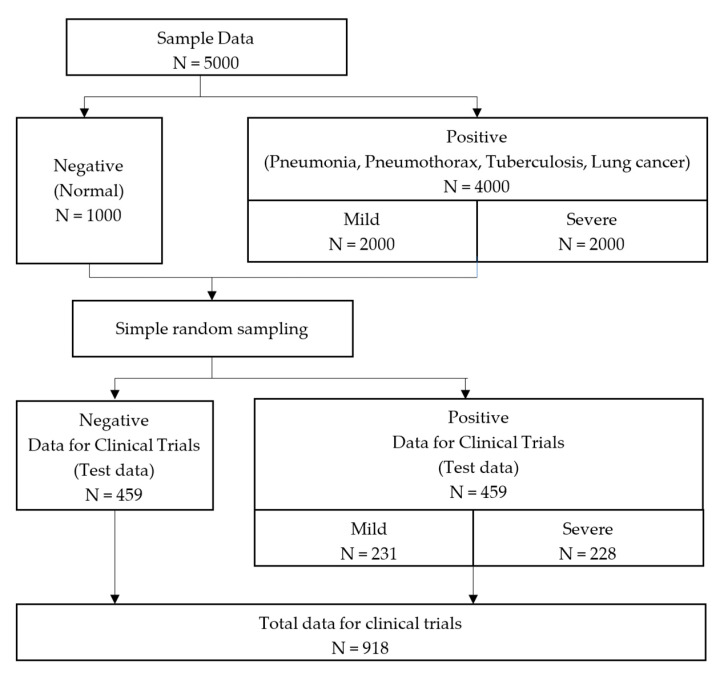
Composition of dataset and test dataset.

**Figure 3:**
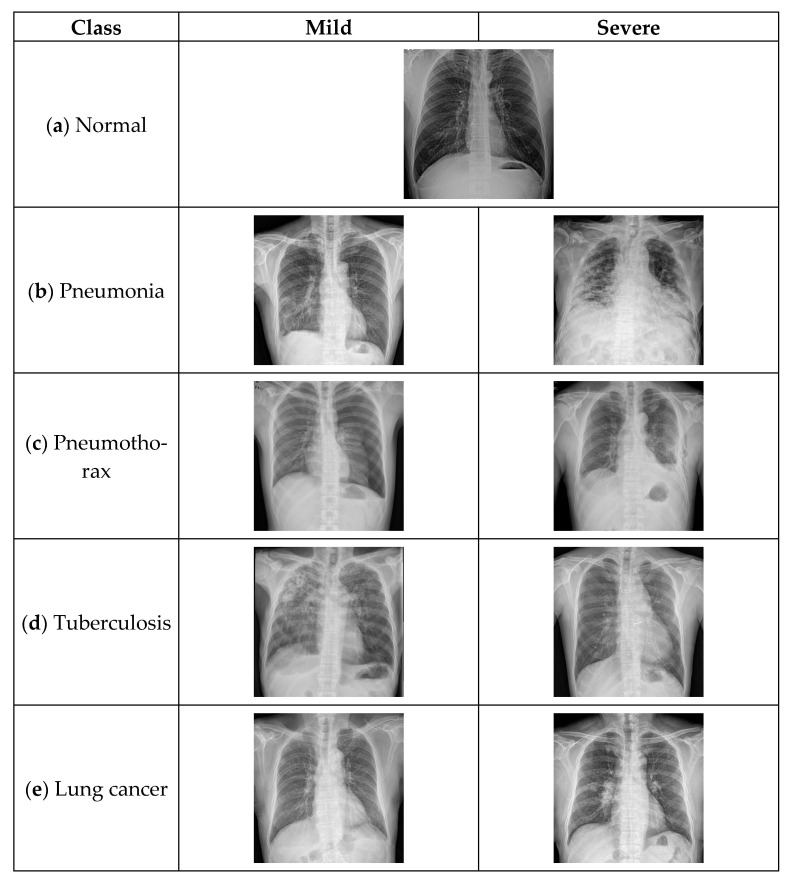
Lung diseases dataset: (**a**) normal; (**b**) pneumonia; (**c**) pneumothorax; (**d**) tuberculosis; (**e**) lung cancer.

The severity of lung cancer was calculated by the eighth edition TNM stage classification for lung cancer, and stage I and II were the mild group and stage III was the severe group [23]. Finally, the Soonchunhyang dataset (n = 5000) was collected as shown in Figure 1, Figure 2 and Figure 3.

### 3.2. Test Dataset

For the number of test datasets, sample size estimation in diagnostic test studies of biomedical informatics [24] Formula (7.5) (p. 198) and ROC Curves in Clinical Chemistry: Uses, Misuses, and Possible Solutions [25] Formula (2) (p. 1123) were used, and the research hypothesis and calculation basis formula at this time are as follows.

(1) $$n=\frac{{\left[Z_{\alpha }\sqrt{V_{H0}\left(\hat{AUC}\right)}+Z_{\beta }\sqrt{V_{H1}\left(\hat{AUC}\right)}\right]}^{2}}{{\left[AUC_{1}-AUC_{0}\right]}^{2}}\;V\left(\hat{AUC}\right)=\left(0.0099\times e^{-\alpha^{2}/2}\right)\times \left(6\alpha^{2}+16\right)\;\alpha =\emptyset^{-1}\left(AUC\right)\times 1.414\;\emptyset^{-1}=Number\;of\;Standard\;Normal\;Distribution\;Type\;I\;error\left(\alpha \right)=0.025\left(One\;sided\right)\;Type\;II\;error\left(\beta \right)=0.1$$

### 3.3. OView-AI Workflow

The OView-AI system is a medical image diagnostic aid software which is based on an abnormal classification model learned by artificial intelligence technology using a chest X-ray image to diagnose a prediction rate of each lesion for pneumonia, pneumothorax, tuberculosis, and lung cancer. Its purpose is to assist a doctor’s diagnosis decision by displaying a prediction rate as a percentage (%).

The principal process is as follows:

- Input a chest X-ray image;
- Adjust image into 1:1 ratio as the pre-processing;
- Classify into normal, pneumonia, pneumothorax, tuberculosis, and lung cancer using the deep learning model;
- Display the classified result as a numerical value (%).

### 3.4. Deep Learning Algorithm

**Figure 4:**
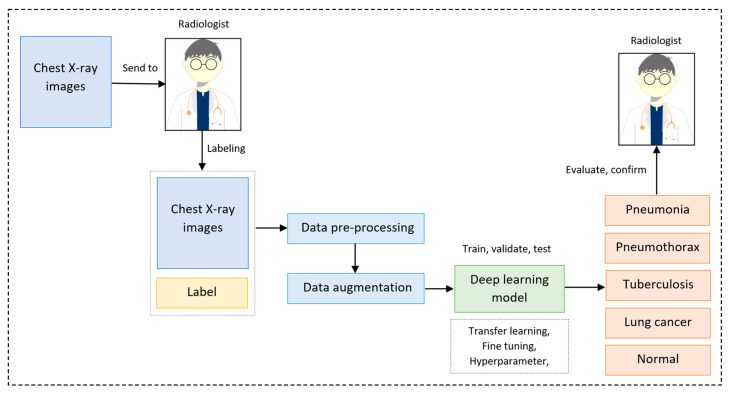
Overall pipeline of multi-classification for lung diseases using transfer learning technique.

As suggested by this literature review, the transfer learning technique has proved its significant performance and lower computational cost. Therefore, in our study, we used the transfer learning technique with EfficientNet B7 [9] with the pre-trained weights of ImageNet trained by Noisy Student [10] as a backbone model. Then, this was followed by our proposed fine-tuned layers and hyperparameters. Figure 4 depicts our overall pipeline of multi-classification for lung diseases using the transfer learning technique.

### 3.5. Deep Learning Model

**Figure 5:**
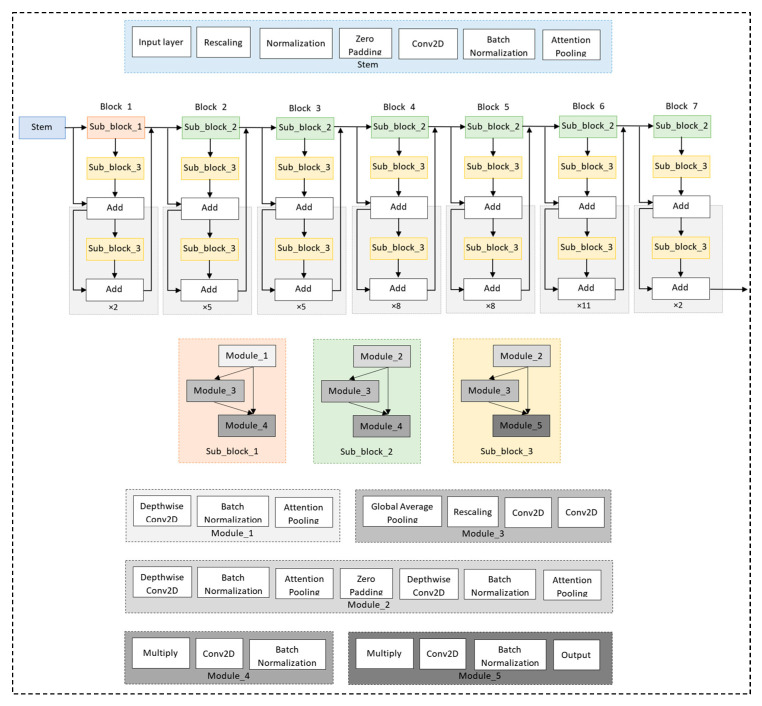
EfficientNet B7 architecture as our backbone model.

The EfficientNet [9] model has proven its efficient learning performance by being principally scaled within three main components: width, depth, and resolution of the model. Compound scaling refers to finding the optimal efficiency by adjusting these components. In our study, we exploited the EfficientNet B7 model with the pre-trained weights of ImageNet trained by Noisy Student [10] as our backbone model, as shown in Figure 5.

EfficientNet B7 takes an input tensor of shape (600 × 600 × 3). Thus, the chest X-ray images were tripled before feeding the data into the based model. The based model (EfficientNet B7) consists of a stem block and seven convolutional blocks. The stem block consists of an input layer, rescaling, normalization, zero padding, conv2D, batch normalization, and attention pooling. Each block consists of three sub-blocks. Block 1 consists of sub-block 1, sub-block 3, and two times their additions. Block 2 to block 7 comprises sub-block 2, sub-block 3, and their additions. Each sub-block is a residual network of three modules among five modules. Module 1 consists of depthwise conv2D, batch normalization, and attention pooling. Module 2 consists of depthwise conv2D, batch normalization, attention pooling, zero padding, depthwise conv2D, batch normalization, and attention pooling. Module 3 consists of global average pooling, rescaling, and two conv2Ds. Module 4 consists of multiply, conv2D, and batch normalization. Module 5 consists of Multiply, conv2D, batch normalization, and output layer. The final layers of Efficient B7 were omitted.

### 3.6. Proposed Fine-Tuned Model

**Figure 6:**
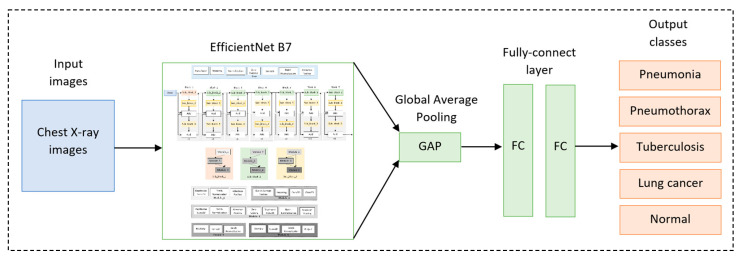
Our proposed fine-tuned model.

The output of the based model (EfficientNet B7) was a 3D tensor. Thus, global average pooling was applied to flatten the features before feeding them into two 512-FCs (fully connected layers). Then, the Softmax function was applied to classify 5 classes (pneumonia, pneumothorax, tuberculosis, lung cancer, and normal). Figure 6 depicts our proposed fine-tuned model.

### 3.7. Hyperparameters

Since the size of EfficientNet B7 (the backbone model) is quite large, the risk of overfitting is also high. To solve this problem, the training was optimized by applying the L2 regularization and dropout layer. L2 regularization was applied to vectors passing through the two FC layers.

(2) $$L_{2}\;regularization\;term=\|w\|=\sqrt{\left(w_{1}^{2}+w_{2}^{2}+w_{3}^{2}+\ldots +w_{n}^{2}\right)}$$

The loss measures how well the model can fit the data, and L2 regularization measures the model complexity. The L2 regularization was calculated by a square root of the sum of the squares of all the feature weights, as shown in Equation (2), before feeding the features into the Softmax function to generate the class probability. The dropout layer with a rate of 0.5 was applied before the last output layer to mit certain neurons.

Since our dataset is imbalanced, the risk of being overly biased to a specific class is also high. To solve this problem, we applied the class weight option during the training. We applied a low weight to a class with a large amount of data and a high weight to a class with a small amount of data. The class weights, $C_{i}$, were calculated as Equation (3).

(3) $$C_{i}=\frac{M}{N\times S_{i}}$$

where N is the number of classes (N=5), M is the total amount of training data (M=73,392), and $S_{i}$ is the total amount of data of class i ($i=\left\{0,\;1,\;2,\;3,\;4\right\}$).

We applied Lookahead [26] to wrap the Nadam optimizer [27]. This optimizer and wrapper prevent our model from local minima, which can solve the overfitting issue. The learning rate scheduling using the warm-up method was also applied. From the first 10% of the epochs, the warm-up learning rate was initial from rate 0 to 0.0001. Then, it stayed as 0.0001 for another 10% of the epochs. After that, it gradually decreased from 0.0001 to 0.00001 using the cosine decay function.

Our ground truth was labeled as the one-hot label, of which the negative was set to 0 and positive was set to 1. We applied the Softmax function as a classifier. Conceptually, the Softmax function generates the class probabilities between 0 and 1, but not exactly 0 and 1. That means that the learning probabilities would not be able to reach the exact 0 and 1. This phenomenon causes an overconfidence issue. To solve this issue, we applied label smoothing [28] with a rate of 0.1 to the loss function. Equation (4) shows the label smoothing ($y_{ls}$) uniform distribution calculation.

(4) $$y_{ls}=\left(1-\alpha \right)\times y_{gt}+\frac{\alpha }{K}$$

where K is the number of label classes (K=5), α is the smoothing rate (α=0.1), and $y_{gt}$ is the one-hot encoded label of ground truth. Table 2 shows our label smoothing distribution.

### 3.8. Environmental Setting

We conducted our experiment on the Linux Ubuntu 18.04 LTS operating system with Intel CPU 19-9940X 3.30 GHz, Nvidia Geforce RTX 3090, RAM 64 GB. The code was written in Python 3.9, Tensorflow 2.5, CUDA 11, and cuDNN 8. The training was set to 50 epochs with a batch size of 16.

## 4. Experimental Results and Discussion

### 4.1. Environmental Results

**Figure 7:**
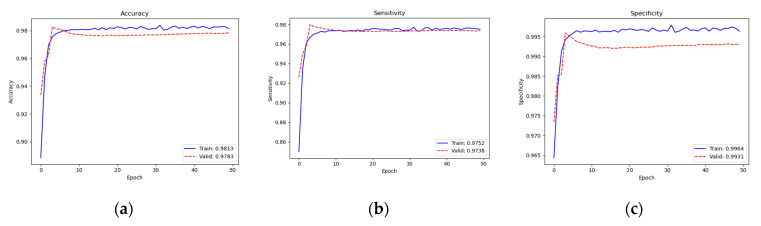
Training and validation performance: (**a**) accuracy; (**b**) sensitivity; (**c**) specificity.

Figure 7 depicts the performance of training and validation. Our learning achieved a training accuracy of 98.13% and a validation accuracy of 97.83, training sensitivity of 97.52% and validation sensitivity of 97.38, and training specificity of 99.64% and validation specificity of 99.31%. We can see that the loss converged well since around epoch 10. After the 10th epoch, the training could be considered to have a stable learning. Thus, 50 epochs were enough to teach our model.

Table 3 shows testing performance by each class in accuracy, sensitivity, and specificity. Our method achieved an accuracy of 98.60% in the normal class, sensitivity of 97.50%, and specificity of 99.83%. Our method achieved an accuracy of 96.91% in the pneumonia class, sensitivity of 95.66%, and specificity of 99.01%. Our method achieved an accuracy of 99.05% in the pneumothorax class, sensitivity of 97.25%, and specificity of 99.15%. Our method achieved an accuracy of 96.88% in the tuberculosis class, sensitivity of 94.98%, and specificity of 99.01%. Our method achieved an accuracy of 95.64% in the lung cancer class, sensitivity of 94.26%, and specificity of 98.04%. On average, our method achieved an accuracy of 97.42%, sensitivity of 95.93%, and specificity of 99.05%.

### 4.2. Discussion

We conducted an inference of the different 918 chest X-ray images for which the data were also provided after de-identification from Soonchunhyang University Hospital. Table 4 shows the inferencing set by each class. The amount of each disease is almost equal (around 111 images), while the amount of healthy (normal) images is 459. Therefore, the total amount of diseases is 459 images equal to the normal set.

The data were tested with our proposed method. The inference result was evaluated and confirmed by professional radiologists of Soonchunhyang University Hospital, as shown in Table 5. Our method achieved a sensitivity of 0.9412, specificity of 0.9935, overall accuracy of 0.9673, and accuracy under the curve (AUC) of 0.9673.

**Figure 8:**
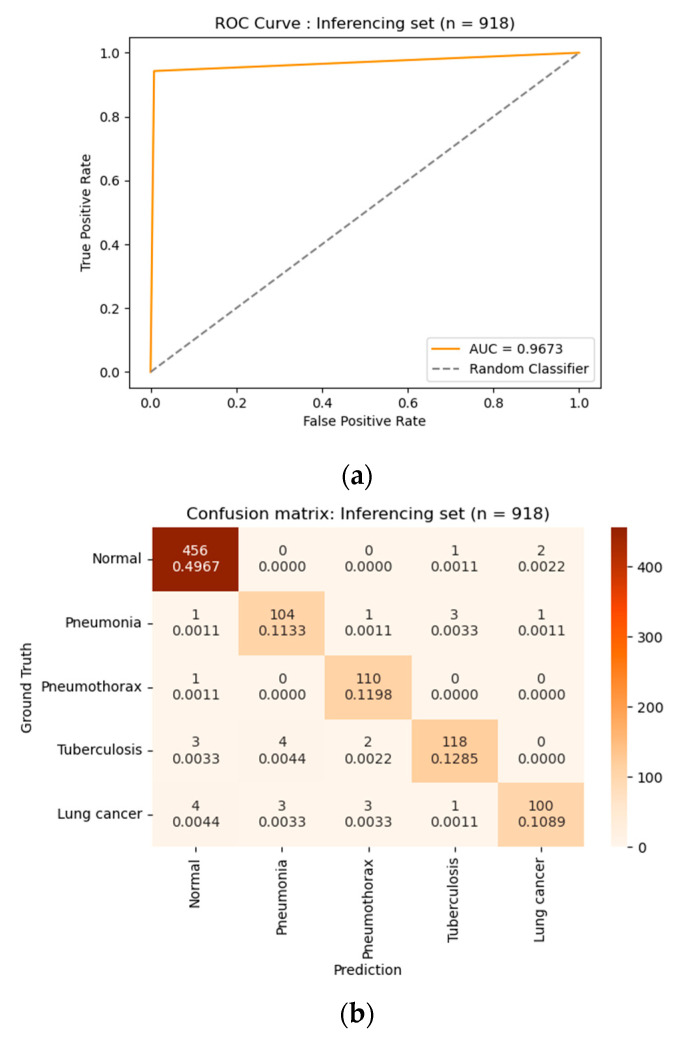
Results of inferencing on 918 images: (**a**) receiver operating characteristic (ROC) curve; (**b**) confusion matrix.

Figure 8 depicts a receiver operating characteristic (ROC) curve and confusion matrix of the 918-image inferences. Our method had a significant prediction and was less confused with other classes. Our method correctly predicted 456 images (49.67%) from 459 images of a normal class (negative) and 432 images (47.05%) from 459 images of a disease class (positive). In each disease, our method correctly predicted 104 images (11.33%) from 110 images of pneumonia class. There were six images (0.66%) confused with other classes. Our method correctly predicted 110 images (11.98%) from 111 images of pneumothorax class. There was one image (0.11%) confused with other classes. Our method correctly predicted 118 images (12.85%) from 127 images of tuberculosis class. There were nine images (0.99%) confused with other classes. Our method correctly predicted 100 images (10.89%) from 111 images of lung cancer class. There were 11 images (1.21%) confused with other classes.

We omitted eight images with the poorest prediction from the inferencing set. Now, the inferencing dataset is made up of 910 images, as shown in Table 6. We removed two images of pneumonia, three images of tuberculosis, and three images of lung cancer.

In total, 910 images were tested with our method. The inference result was evaluated and confirmed by professional radiologists of Soonchunhyang University Hospital, as shown in Table 7. Our method achieved a sensitivity of 0.9468, specificity of 0.9934, overall accuracy of 0.9703, and accuracy under the curve (AUC) of 0.9701.

**Figure 9:**
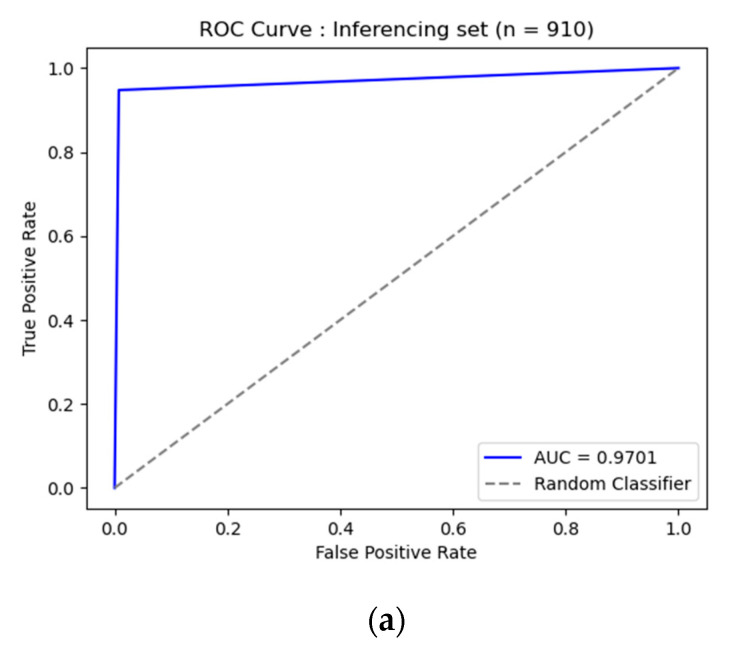

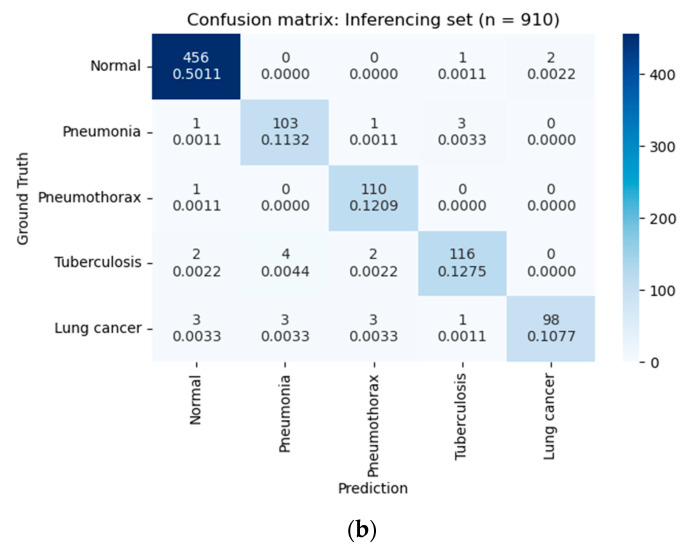
Results of inferencing on 910 images: (**a**) receiver operating characteristic (ROC) curve; (**b**) confusion matrix.

Figure 9 depicts a receiver operating characteristic (ROC) curve and confusion matrix of the 910-image inferences. Our method had a significant prediction and was less confused with other classes. Our method correctly predicted 456 images (50.11%) from 459 images of a normal class (negative) and 427 images (46.93%) from 451 images of a disease class (positive). In each disease, our method correctly predicted 103 images (11.32%) from 108 images of pneumonia class. There were four images (0.44%) confused with other classes. Our method correctly predicted 110 images (11.98%) from 111 images of pneumothorax class. There was one image (0.11%) confused with other classes. Our method correctly predicted 116 images (12.75%) from 124 images of tuberculosis class. There were eight images (0.88%) confused with other classes. Our method correctly predicted 98 images (10.77%) from 108 images of lung cancer class. There were 10 images (1.10%) confused with other classes.

Table 8 summarizes and compares the inferences of the 918-image set and 910-image set. With the 918-image set, our method achieved an overall accuracy of 96.73%, AUC of 96.73%, and 2.6282 s per inference. With the 918-image set, our method performed better and achieved an overall accuracy of 97.03%, AUC of 97.01%, and 2.6275 s per inference.

## 5. Conclusions

We proposed a classification of four lung diseases and healthy lungs using a deep learning method. Our proposed fine-tuned deep learning model, which composed of EfficientNet B7 as the backbone, fine-tuned layers, and hyperparameters, showed itself to present a significant performance in multi-lung infections. Chest X-ray images were collected from Soonchunhyang University Hospital after de-identification from May 2022 to July 2022. Our method achieved an average test accuracy, sensitivity, and specificity of 97.42%, 95.93%, and 99.05%, respectively. Additionally, we conducted an inference on other different 910 chest X-ray images. The AUC confidence interval (95%CI) of diagnostic results in the OView-AI system (diagnostic supporting software detects normal lung cancer, pneumonia, pulmonary tuberculosis, and pneumothorax using chest X-ray images) was 97.01%. Moreover, the sensitivity and specificity of the OView-AI system were 94.68% and 99.34%, respectively.

The inference experiment was evaluated and confirmed by professional radiologists of Soonchunhyang University Hospital. The inferencing results expressed that our method had a highly correct prediction of healthy lungs (negative class) and less confused other diseases (positive class). Among diseases, our method had the highest correct prediction of the pneumothorax class. Our findings demonstrated the potential to fasten the clinical workflow and facilitate the early screening stage for multi-chest infections as the computer-aided application (OView-AI System), which supports clinical decision making.

## Figures and Tables

**Table 1:** Related papers conducted on classification for lung diseases.

Paper	Disease	Class	Model	Result
Asif et al. (2022) [11]	COVID-19, viral pneumonia, and normal	3	Transfer learning (VGG-16)	Accuracy = 97.84%
Hamwi et al. (2022) [12]	COVID-19, and normal	2	Transfer learning (merged model of VGG-16 and DenseNet-201)	Accuracy = 99.73%
Hong et al. (2021) [13]	Pneumonia, pneumothorax, tuberculosis, and normal	4	Transfer learning (EfficienNet B7)	Accuracy = 96.10%
Hu et al. (2022) [14]	COVID-19, non-COVID-19 pneumonia, and normal	3	Train from scratch (VGG-16)	AUC-ROC = 97.80%
Kim et al. (2022) [15]	Pneumonia, pneumothorax, tuberculosis, and normal	4	Transfer learning (EfficientNet v2M)	Accuracy = 82.20%
Malik et al. (2022) [16]	Pneumonia, pneumothorax, tuberculosis, COVID-19, and normal	5	Train from scratch (residual network and dilated convolution)	Accuracy = 99.39%
Manalakis et al. (2021) [17]	COVID-19, tuberculosis, pneumonia, and normal	4	Transfer learning (merged model of DenseNet-121 and ResNet-50)	AUC-ROC = 95.00%
Shamrat et al. (2022) [18]	COVID-19, effusion, tuberculosis, pneumonia, lung opacity, mass, nodule, pneumothorax, pulmonary fibrosis, and normal	10	Transfer learning (VGG-16)	Accuracy = 98.89%
Showkatian et al. (2022) [19]	Tuberculosis and normal	2	Train CNN from scratch (CNN)	Accuracy = 87.00%
Xu et al. (2022) [20]	Tuberculosis (TB), diseases but non-TB, and normal	3	Train from scratch (DenseNet)	Accuracy = 99.10%

**Table 2:** Our label smoothing distribution.

Class State	Ground Truth Label $\left(y_{gt}\right)$	Label Smoothing $\left(y_{ls}\right)$
Negative	0	0.02
Positive	1	0.92

**Table 3:** Testing performance by each class.

Class	Accuracy	Sensitivity	Specificity
Normal	98.60%	97.50%	99.83%
Pneumonia	96.91%	95.66%	99.21%
Pneumothorax	99.05%	97.25%	99.15%
Tuberculosis	96.88%	94.98%	99.01%
Lung cancer	95.64%	94.26%	98.04%
Average	97.42%	95.93%	99.05%

**Table 4:** Inferencing dataset collected from Soonchunhyang University Hospital.

Class	Inferencing Set		Inferencing Set
Normal	459	Healthy	459
Pneumonia	110	Diseases	459
Pneumothorax	111		
Tuberculosis	127		
Lung cancer	111		
Total	918		918

**Table 5:** Evaluation result of our method on the 918-image inferences.

	Equation	Proportion Estimate	Confidence Interval	
			Lower	Upper
Sensitivity	*TP/(TP + FN)*	0.9412	0.9197	0.9627
Specificity	*TN/(FP + TN)*	0.9935	0.9861	1.0008
Likelihood ratio +	*TP/(TP + FN)/(FP/FP + TN))*	144.0000	46.6037	444.9436
Likelihood ratio −	*FN/(TP + FN)/(TN/FP + TN))*	0.0592	0.0411	0.0854
False positive rate	*FP/(FP + TN)*	0.0065	−0.0008	0.0139
False negative rate	*FN/(TP + FN)*	0.0588	0.0373	0.0798
Prob of disease	*(TP + FN)/(TP + FP + FN + TN)*	0.5000	0.4677	0.5323
Positive predictive value	*TP/(TP + FP)*	0.9931	0.9853	1.0009
p (pos test wrong)	*FP/(TP + FP)*	0.0069	−0.0009	0.0147
Negative predictive value	*TN/(FN + TN)*	0.9441	0.9236	0.9646
p (neg test wrong)	*FN/(FN + TN)*	0.0559	0.0354	0.0764
p (test positive)	*(TP + FP)/(TP + FP + FN + TN)*	0.4739	0.4416	0.5062
p (test negative)	*(FN + TN)/(TP + FP + FN + TN)*	0.5261	0.4938	0.5584
Overall accuracy	*(TP + TN)/(TP + FP + FN + TN)*	0.9673	0.9558	0.9788
AUC	*(Sensitivity + Specificity)/2*	0.9673	0.9555	0.9791

*TP*, *TN*, *FP*, and *FN* are true positive, true negative, false positive, and false negative, respectively.

**Table 6:** Inferencing dataset collected from Soonchunhyang University Hospital.

Class	Inferencing Set		Inferencing Set
Normal	459	Healthy	459
Pneumonia	108	Diseases	451
Pneumothorax	111		
Tuberculosis	124		
Lung cancer	108		
Total	910		910

**Table 7:** Inferencing dataset collected from Soonchunhyang University Hospital.

	Equation	Proportion Estimate	Confidence Interval	
			Lower	Upper
Sensitivity	*TP/(TP + FN)*	0.9468	0.9261	0.9675
Specificity	*TN/(FP + TN)*	0.9934	0.9861	1.0008
Likelihood ratio +	*TP/(TP + FN)/(FP/FP + TN))*	144.5425	46.7805	446.6075
Likelihood ratio −	*FN/(TP + FN)/(TN/FP + TN))*	0.0536	0.0363	0.0791
False positive rate	*FP/(FP + TN)*	0.0066	−0.0008	0.0139
False negative rate	*FN/(TP + FN)*	0.0532	0.0325	0.0733
Prob of disease	*(TP + FN)/(TP + FP + FN + TN)*	0.4961	0.4636	0.5287
Positive predictive value	*TP/(TP + FP)*	0.9930	0.9852	1.0009
p(pos test wrong)	*FP/(TP + FP)*	0.0070	−0.0009	0.0148
Negative predictive value	*TN/(FN + TN)*	0.9499	0.9304	0.9694
p(neg test wrong)	*FN/(FN + TN)*	0.0501	0.0306	0.0696
p(test positive)	*(TP + FP)/(TP + FP + FN + TN)*	0.4730	0.4406	0.5055
p(test negative)	*(FN + TN)/(TP + FP + FN + TN)*	0.5270	0.4945	0.5594
Overall accuracy	*(TP + TN)/(TP + FP + FN + TN)*	0.9703	0.9593	0.9813
AUC	*(Sensitivity + Specificity)/2*	0.9701	0.9588	0.9815

*TP*, *TN*, *FP*, and *FN* are true positive, true negative, false positive, and false negative, respectively.

**Table 8:** Inference overall accuracy, accuracy under the curve, and time.

Inference	Overall Accuracy	AUC	Time per Inference
918 images	96.73%	96.73%	2.6282 s
910 images	97.03%	97.01%	2.6275 s

## Data Availability

No new data were created in this study. Data sharing is not applicable.
